# Dataset from the dynamic shake-table test of a full-scale unreinforced clay-masonry building with flexible timber diaphragms

**DOI:** 10.1016/j.dib.2018.03.047

**Published:** 2018-03-16

**Authors:** Stylianos Kallioras, Gabriele Guerrini, Umberto Tomassetti, Simone Peloso, Francesco Graziotti

**Affiliations:** a UME Graduate School, IUSS Pavia, Piazza della Vittoria 15, 27100 Pavia, Italy; b Department of Civil Engineering and Architecture (DICAr), University of Pavia, via Ferrata 3, 27100 Pavia, Italy; c European Centre for Training and Research in Earthquake Engineering (EUCENTRE), via Ferrata 1, 27100 Pavia, Italy

## Abstract

This paper provides information related to the sensor measurements obtained from an unreinforced masonry building subjected to incremental dynamic shake-table tests at the EUCENTRE facilities in Pavia, Italy. These tests provide a unique data set that captures at full scale the in-plane and out-of-plane behavior of unreinforced masonry walls, and the influence of flexible diaphragms on the dynamic global response of a complete building. The authors made this information available to assist in the development of analytical and numerical models, necessary to estimate the dynamic response and the engineering parameters for the performance-based seismic assessment of unreinforced masonry buildings. All recorded data (acceleration and displacement time histories) and the videos of the tests can be requested online on the EUCENTRE repository at the URL www.eucentre.it/nam-project referring to EUC-BUILD-2. For further interpretation of the sensor recordings, and for a detailed discussion on the seismic performance of the building specimen, the reader is referred to the article entitled “Experimental seismic performance of a full-scale unreinforced clay-masonry building with flexible timber diaphragms” (Kallioras et al., 2018) [1].

**Specifications table**

**Table:** 

Subject area	*Engineering*
More specific subject area	*Structural engineering, Earthquake engineering*
Type of data	*Acceleration and displacement time histories*
How data was acquired	*Ground shaking of increasing intensity was applied to the building base, while random vibration tests were performed to monitor the evolution of the system dynamic properties at each testing step. The specimen was densely instrumented with accelerometers, wire potentiometers, linear potentiometers, and a three-dimensional motion-capture system that recorded the response of various structural elements*
Data format	*.txt files and .mat files with filtered and processed time histories*
Experimental factors	*The specimen represented a typical unreinforced masonry detached house of the Groningen region of the Netherlands*
Experimental features	*Incremental unidirectional dynamic shake-table tests were performed up to near-collapse conditions of the building, using input ground motions compatible with induced-seismicity scenarios for the Groningen region of the Netherlands*
Data source location	*The tests were carried out at the laboratory facilities of the European Centre for Training and Research in Earthquake Engineering (EUCENTRE) based in Pavia, Italy*
Data accessibility	*All recorded data (acceleration and displacement time histories) and the videos of the tests can be requested online on the EUCENTRE repository at the URL*www.eucentre.it/nam-project*referring to EUC-BUILD-2*.

**Value of the data**

• The paper describes a comprehensive testing campaign that could serve as a benchmark in the field of laboratory testing of structures.
• The instrument measurements provide detailed information about the building dynamic response and could be employed for the calibration of analytical and numerical models.
• The recordings of the optical acquisition system could be used by researchers to establish relationships between local and global damage indicators of masonry wall structures.
• The obtained measurements could be further analyzed to evaluate the effect of flexible diaphragms in the dynamic response of masonry buildings.
• The test outcomes could be used to validate simplified methods to estimate earthquake-induced deformation demands with the focus on unreinforced masonry buildings.

## 1 Experimental design, materials and methods

**Fig. 1:**
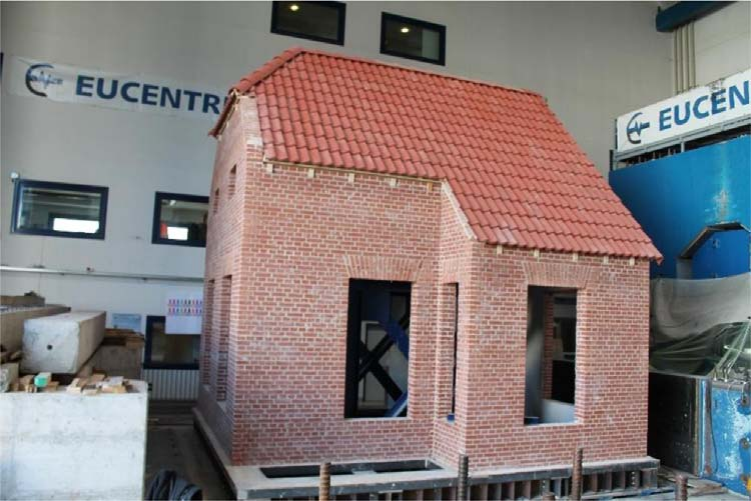
Full-scale building specimen at the EUCENTRE laboratory facilities.

A unidirectional shake-table test was conducted on a full-scale prototype of a clay-brick unreinforced masonry (URM) detached house, featuring typical details of the pre-1940s construction practice of the Groningen region in the Netherlands (Fig. 1). The prototype consisted of double-wythe clay-brick unreinforced masonry walls, not detailed for seismic resistance, with large openings and a reentrant corner which caused discontinuities along the perimeter walls. The floor system of timber beams and planks provided a flexible diaphragm. The roof was characterized by a very steep pitch; its structure consisted of timber trusses, purlins and boards. The two façades perpendicular to the shaking direction included two typical gable geometries.

The specimen was subjected to incremental dynamic excitations, with input motions representative of induced seismicity scenarios for the Groningen region, characterized by smooth response spectra and short significant duration. The ground motions were progressively scaled in amplitude to achieve the desired demand intensities up to the near-collapse state of the building. Random vibration tests were also performed to monitor the evolution of the system dynamic properties at each testing step.

The geometric characteristics of the building specimen, the construction details, the material properties, the instrumentation plan, the testing protocol, and the major observations from the tests are described in detail in [1]. Therein, the instrument recordings have been employed to link engineering demand parameters to the attainment of significant performance limits states for the assessment of the seismic behavior of the prototype building. Further information about the materials used to construct the specimen and the results of the companion tests on wall components can be found in [2].

## 2 Data

The specimen was densely instrumented with sensors that recorded the dynamic response at various locations. All recorded acceleration and displacement time histories and the videos of the tests are available upon request at the following URL: www.eucentre.it/nam-project.

### 2.1 Data acquisition: accelerometers and potentiometers

The instrumentation consisted of 38 accelerometers, 21 wire potentiometers, and 37 linear potentiometers, installed on the building to capture its structural response during the dynamic tests. The majority of the sensors was mounted in the shaking direction, while some were also oriented transversely or vertically. The instrumentation plan is illustrated in Figs. 9 and 10 of Ref. [1].

Data obtained from accelerometers and potentiometers are provided in twelve *.txt* files, named after the corresponding shake-table test, as defined in Table 1. Each file is a two-dimensional matrix of 144 columns, where each column contains the time history of a measured or derived physical quantity. The lines of the *.txt* files correspond to individual instants of the time series.

**Table 1:** Accelerometer and potentiometer data: file names.

**Test No.**	**Test name**	**Data file name**	**Matrix sizes (rows No. × columns No.)**
1	SC1 - 25%	#03–25%-SC1–024	5121 × 144
2	SC1 - 50%	#05–50%-SC1–047	5121 × 144
3	SC1 - 100%	#07–100%-SC1–094	5121 × 144
4	SC1 - 150%	#09–150%-SC1–141	5121 × 144
5	SC2 - 50%	#13–50%-SC2–076	7681 × 144
6	SC2 - 100%	#15–100%-SC2–152	7681 × 144
7	SC2 - 150%	#20–150%-SC2–228	7681 × 144
8	SC2 - 200%	#22–200%-SC2–304	7681 × 144
9	SC2 - 250%	#24–250%-SC2–380	7681 × 144
10	SC2 - 300%	#30–300%-SC2–456	7681 × 144
11	SC2 - 400%	#36–400%-SC2–608	7681 × 144
12	SC2 - 200%-A^a^	#41–200%-SC2–304	7681 × 144

a A: test simulating an aftershock

Table 2 lists the content of the first 124 columns of each matrix, corresponding to quantities directly measured by the sensors: number of column; sensor identification number; recorded degree of freedom; measurement units; brief description of measured quantity and location of the instrument; mass attributed to accelerometer location (Table 2). All acceleration and displacement recordings were filtered using a quadratic low-pass filter set to a frequency of 50 Hz. The data are organized in each file as follows:

i. Column 1 contains the time at a sampling rate 256 Hz;
ii. Columns 2–58 contain the acceleration time histories recorded by the 38 accelerometers, corresponding to 52 degrees of freedom. Note that five in-between channels, shown in grey in the table, were offline. Acceleration recordings are provided in units of g;
iii. Columns 59–124 contain the displacement time histories measured by wire and linear potentiometers. Note that eight in-between channels, shown in grey in the table, were offline. Displacement measurements are expressed in units of mm.

**Table 2:** Accelerometer and potentiometer recordings: matrix columns 1 to 124. Letters indicate the type of measuring instrument: A, accelerometer; WP, wire potentiometer; P, potentiometer.

**Col. No.**	**Sensor ID No.**	**Rec. DOF**	**UM**	**Assocd. mass (in x dir.) [kg]**	**Measured quantity - Instrument location**
1	-	-	[s]	-	Time
2	A 1	x	[g]	146	Floor diaphragm acceleration (S-W corner, +2900)
3		y	[g]	-	
4		z	[g]	-	
5	A 2	x	[g]	220	Floor diaphragm accel. (S-E corner, +2900)
6	Offline	-	-	-	^*^for y component of A 2, see column No.53
7	A 2	z	[g]	-	Floor diaphragm accel. (S-E corner, +2900)
8	Offline	-	-	-	^*^for x component of A 3, see column No.54
9	Offline	-	-	-	^*^for y component of A 3, see column No.54
10	A 3	z	[g]	-	Floor diaphragm accel. (N-E corner, +2900)
11	A 4	x	[g]	220	Floor diaphragm accel. (N-W corner, +2900)
12		y	[g]	-	
13		z	[g]	-	
14	A 5	x	[g]	240	Floor diaphragm accel. (West protruding corner, +2900)
15	A 6	x	[g]	315	Floor diaphragm accel. (centre of mass, +2900)
16		y	[g]	-	
17		z	[g]	-	
18	A 7	x	[g]	451	Roof ridge beam accel. (South end, +6074 mm)
19		y	[g]	-	
20		z	[g]	-	
21	A 8	x	[g]	548	Roof ridge beam accel. (North end, +6074 mm)
22		y	[g]	-	
23		z	[g]	-	
24	Offline	-	-	-	^*^for z component of A 9, see column No.56
25	A 10	x	[g]	1554	South wall accel. (S-W corner, +2900)
26	A 11	x	[g]	1789	South wall accel. (midspan, +2900)
27	A 12	x	[g]	921	South wall accel. (S-E corner, +2900)
28	A 13	x	[g]	1154	North wall accel. (N-E corner, +2900)
29	A 14	x	[g]	1203	North wall accel. (midspan, +2900)
30	A 15	x	[g]	1611	North wall accel. (N-W corner, +2900)
31	A 16	x	[g]	266	Floor diaphragm accel. (South midspan, +2900)
32	A 17	x	[g]	275	Floor diaphragm accel. (North midspan, +2900)
33	A 18	x	[g]	910	South wall mid-height accel. (midspan, +1450 mm)
34	A 19	x	[g]	761	South gable wall accel. (S/W corner, +3815 mm)
35	A 20	x	[g]	671	South gable wall top accel. (+6031 mm)
36	A 21	x	[g]	584	North wall mid-height accel. (midspan, +1430 mm)
37	A 22	x	[g]	145	North clipped gable wall mid-height accel. (midspan, +3815 mm)
38	A 23	x	[g]	1146	North clipped gable wall top accel. (midspan, +4847 mm)
39	A 24	x	[g]	4755	Foundation beam accel. (West, +0.000)
40	A 25	x	[g]	4034	Foundation beam accel. (East, +0.000)
41	A 26	x	[g]	493	North clipped gable wall mid-height acc. (N-E corner, +3815 mm)
42	A 27	x	[g]	-	Steel frame accel. (N-W column, +2900)
43	A 28	x	[g]	-	Steel frame top accel. (North side)
44	A 29	x	[g]	1456	East wall accel. (Southern side, +2900)
45	A 30	x	[g]	1986	East wall accel. (Northern side, +2900)
46	A 31	x	[g]	891	West wall accel. (protruding corner, +2900 mm)
47	A 32	x	[g]	1202	West wall accel. (reentrant corner, +2900 mm)
48	A 33	x	[g]	493	Roof/Wall plate accel. (S-W corner, +2900)
49	A 34	x	[g]	286	Roof/Wall plate accel. (S-E corner, +3840)
50	A 35	x	[g]	250	Roof/Wall plate accel. (N-E corner, +3840)
51	A 36	x	[g]	250	Roof/Wall plate accel. (N-W corner, +3840)
52	A 37	x	[g]	875	South gable wall accel. (S-E corner, + 3815 mm)
53	A 2	y	[g]	-	Floor diaphragm accel. (S-E corner, +2900)
54	A 3	x	[g]	224	Floor diaphragm accel. (N-E corner, +2900)
55		y	[g]	-	
56	A 9	z	[g]	-	Roof ridge beam accel. (midspan, +6074)
57	A 38	x	[g]	596	North clipped gable wall mid-height acc. (N-W corner, + 3815 mm)
58	Offline	-	-	-	-
59	WP 1	x	[mm]	-	South wall mid-height deflection (w.r.t. the steel frame, +1474 mm)
60	WP 2	x	[mm]	-	North wall mid-height defl. (w.r.t. the steel frame, +1428 mm)
61	WP 3	x	[mm]	-	South gable wall top defl. (w.r.t. the steel frame, +5800 mm)
62	WP 4	x	[mm]	-	North clipped gable wall top defl. (w.r.t. the steel fr., +4824 mm)
63	WP 5	x	[mm]	-	Roof ridge beam displacement (w.r.t. the steel frame, +6074 mm)
64	WP 6	x	[mm]	-	Floor diaphragm longitudinal deformation (along the East wall)
65	WP 7	x	[mm]	-	Floor diaphragm longitudinal def. (along the West wall)
66	WP 8	NW-SE	[mm]	-	Floor diaphragm shear def. (along the N-W to S-E diagonal)
67	WP 9	NE-SW	[mm]	-	Floor diaphragm shear def. (along the N-E to S-W diagonal)
68	WP 46	diagonal	[mm]	-	East squat pier def. (along the bottom/right to top/left diagonal)
69	WP 47	diagonal	[mm]	-	East squat pier def. (along the bottom/left to top/right diagonal)
70	WP 51	diagonal	[mm]	-	East wall top def. (along the bottom/right to top/left diagonal)
71	WP 52	diagonal	[mm]	-	East wall top def. (along the bottom/left to top/right diagonal)
72	Offline	-	-	-	-
73	P 10	x	[mm]	-	Floor diaph. displ. (w.r.t. the steel frame, S-W corner, +2900 mm)
74	P 11	y	[mm]	-	
75	P 12	x	[mm]	-	Floor diaph. displ. (w.r.t. the steel frame, South, +2900 mm)
76	P 13	x	[mm]	-	Floor diaph. displ. (w.r.t. the steel fr., South midspan, +2900 mm)
77	P14	x	[mm]	-	Floor diaph. displ. (w.r.t. the steel frame, S-E corner, +2900 mm)
78	P 15	y	[mm]	-	
79	P 16	x	[mm]	-	Floor diaph. displ. (w.r.t. the steel frame, N-E corner, +2900 mm)
80	P 17	y	[mm]	-	
81	P 18	x	[mm]	-	Floor diaph. displ. (w.r.t. the steel fr., North midspan, +2900 mm)
82	P 19	x	[mm]	-	Floor diaph. displ. (w.r.t. the steel frame, N-W corner, +2900 mm)
83	P 20	y	[mm]	-	
84	P 21	x	[mm]	-	South wall defl. (w.r.t. the floor diaph., S-W corner, +2900 mm)
85	P 22	x	[mm]	-	South wall defl. (w.r.t. the floor diaph., midspan, +2900 mm)
86	P 23	x	[mm]	-	South wall defl. (w.r.t. the floor diaph., S-E corner, +2900 mm)
87	P 24	x	[mm]	-	North wall defl. (w.r.t. the floor diaph., N-E corner, +2900 mm)
88	P 25	x	[mm]	-	North wall defl. (w.r.t. the floor diaph., midspan, +2900 mm)
89	P 26	x	[mm]	-	North wall defl. (w.r.t. the floor diaph., N-W corner, +2900 mm)
90	P 27	y	[mm]	-	East wall top displ. (w.r.t. the steel frame, South side, +3755 mm)
91	P 28	x	[mm]	-	
92	P 29	y	[mm]	-	East wall top displ. (w.r.t. the steel frame, North side, +3755 mm)
93	P 30	x	[mm]	-	
94	P 31	y	[mm]	-	West wall top displ. (w.r.t. the steel frame, North side, +3755 mm)
95	P 32	x	[mm]	-	
96	P 33	x	[mm]	-	Roof ridge beam displ. (w.r.t. the top of South gable, +5710 mm)
97	P 34	x	[mm]	-	Roof truss (East rafter) displ. (w.r.t. the North clipped gable)
98	P 35	x	[mm]	-	Roof truss (West rafter) displ. (w.r.t. the North clipped gable)
99	P 36	x	[mm]	-	East wall sliding (w.r.t. the foundation beam)
100	P 37	x	[mm]	-	Foundation sliding (w.r.t. the shake table, East side)
101	P 38	x	[mm]	-	West innermost wall sliding (w.r.t. the foundation beam)
102	P 39	x	[mm]	-	Foundation sliding (w.r.t. the shake table, West side)
103	Offline	-	-	-	-
104	P 41	x	[mm]	-	North clipped gable top displ. (w.r.t. the hip wall plate, midspan)
105	WP 42	z	[mm]	-	East southernmost pier uplift
106	WP 43	x	[mm]	-	East squat pier horizontal deformation (top)
107	WP 44	z	[mm]	-	East squat pier vertical deformation (left)
108	WP 45	z	[mm]	-	East squat pier vertical deformation (right)
109	WP 48	x	[mm]	-	East wall top horizontal deformation (top)
110	WP 49	z	[mm]	-	East wall top vertical deformation (left)
111	WP 50	z	[mm]	-	East wall top vertical deformation (right)
112	P 53	x	[mm]	-	Floor diaph. displ. (w.r.t. the West wall, protr. corner, +2900 mm)
113	P 54	x	[mm]	-	Roof/Wall plate sliding (w.r.t. the top of East wall, +3755 mm)
114	Offline	-	-	-	-
115	P 56	x	[mm]	-	Roof truss (East rafter) displ. (w.r.t. the South gable wall)
116	WP 57	x	[mm]	-	East squat pier horizontal def. (mid-height)
117	P 55	x	[mm]	-	Roof truss (West rafter) displ. (w.r.t. the South gable wall)
118	P 40	x	[mm]	-	Shake table displ. (w.r.t. the laboratory floor)
119	Offline	-	-	-	-
120	Offline	-	-	-	-
121	Offline	-	-	-	-
122	Offline	-	-	-	-
123	P 58	x	[mm]	-	Roof ridge beam displ. (w.r.t. the steel frame, +6074)
124	Offline	-	-	-	-

Table 3 describes the quantities provided in columns 125–144 of each.txt file, which were not directly measured by the acquisition system, but were derived after post-processing. Quantities such as inertia forces (e.g., base shear and gables-roof inertia forces), interstorey drift ratios, and shear deformations were computed after the assumptions mentioned in sections 5.3 and 5.5 of Ref. [1]. Accelerations and forces are provided in units of g and kN, respectively; displacements are given in mm, and shear deformations are expressed in percentage.

**Table 3:** Accelerometer and potentiometer derived data: matrix columns 125–144.

**Col. No.**	**Recorded / Computed quantity**	**UM**	**Description**
125	Base accel. (*x* dir.), *a*_*g*_	[g]	Average of col. 39 and 40
126	Roof ridge accel. (*x* dir.), *a*_*R*_	[g]	Average of col. 18 and 21
127	East wall floor displ. (*x* dir., +2904 mm), *Δ*_1*,E*_	[mm]	Average of col. 77 and 79
128	West wall floor displ. (*x* dir., +2904 mm), *Δ*_1*,W*_	[mm]	Average of col. 73 and 82
129	Average floor displ. (*x* dir., +2904 mm), *Δ*_1*,AV*_	[mm]	Average of col. 127 and 128
130	East wall top displ. (*x* dir., +3755 mm), *Δ*_*t,E*_	[mm]	Average of col. 91 and 93
131	West wall top displ. (*x* dir., +3755 mm), *Δ*_*t,W*_	[mm]	Equal to col. 95
132	Average wall top displ. (*x* dir., +3755 mm), *Δ*_*t,AV*_	[mm]	Average of col. 130 and 131
133	East wall interstorey drift ratio (*x* dir.), *θ*_1*,E*_	[%]	Ratio of col. 127 and the distance of the floor diaphragm from the base, *h*_1_ = 2904 mm
134	West wall interstorey drift ratio (*x* dir.), *θ*_1*,W*_	[%]	Ratio of col. 128 and the distance of the floor diaphragm from the base, *h*_1_ = 2904 mm
135	Average interstorey drift ratio (*x* dir.), *θ*_1*,AV*_	[%]	Average of col. 133 and 134
136	Roof diaphragm shear def. (East side), *γ*_*R,E*_	[%]	Ratio of difference between col. 123 and col. 130, and the inclined length of the roof, *l*_*R*_ = 3150 mm
137	Roof diaphragm shear def. (West side), *γ*_*R,W*_	[%]	Ratio of difference between col. 123 and col. 131, and the inclined length of the roof, *l*_*R*_ = 3150 mm
138	Average diaphragm shear def., *γ*_*R,AV*_	[%]	Average of col. 136 and 137
139	East wall base shear (*x* dir.), *V*_*b,E*_	[kN]	Inertia force of East wall plus half of inertia force of N/S walls
140	West wall base shear (*x* dir.), *V*_*b,W*_	[kN]	Inertia force of East wall plus half of inertia force of N/S walls
141	Overall base shear (*x* dir.), *V*_*b*_	[kN]	Sum of the products of each accelerometer reading with the associated mass
142	Gables-roof assembly inertia force (*x* dir.), *F*_*R*_	[kN]	Sum of the products of each accelerometer reading with the associated mass above the floor level
143	Overall base shear (*x* dir.), *V*_*b*_*’* (inertia force without non-oscillatory mass)	[kN]	Col. 141 minus the sum of products of col. 39 and 40 with masses 4755.2 and 4033.5 kg, respectively
144	Floor diaphragm in-plane shear deformation, *γ*_*f*_	[%]	–
145	East wall base shear (*x* dir.), *V*_*b,E*_*’* (inertia force without non-oscillatory mass)	[kN]	Column 139 minus the product of column 40 times mass 4033.5 kg
146	West wall base shear (*x* dir.), *V*_*b,W*_*’* (inertia force without non-oscillatory mass)	[kN]	Column 140 minus the product of column 39 times mass 4755.2 kg
147	Inertia force for top half portion of gables-roof assembly (*x* dir.), *F*_*R*_*’*	[kN]	Sum of the product of columns 35 and 38 times masses 1327.2 kg and 872.8 kg, respectively

### 2.2 Data acquisition: 3D optical motion-capture system

**Fig. 2:**
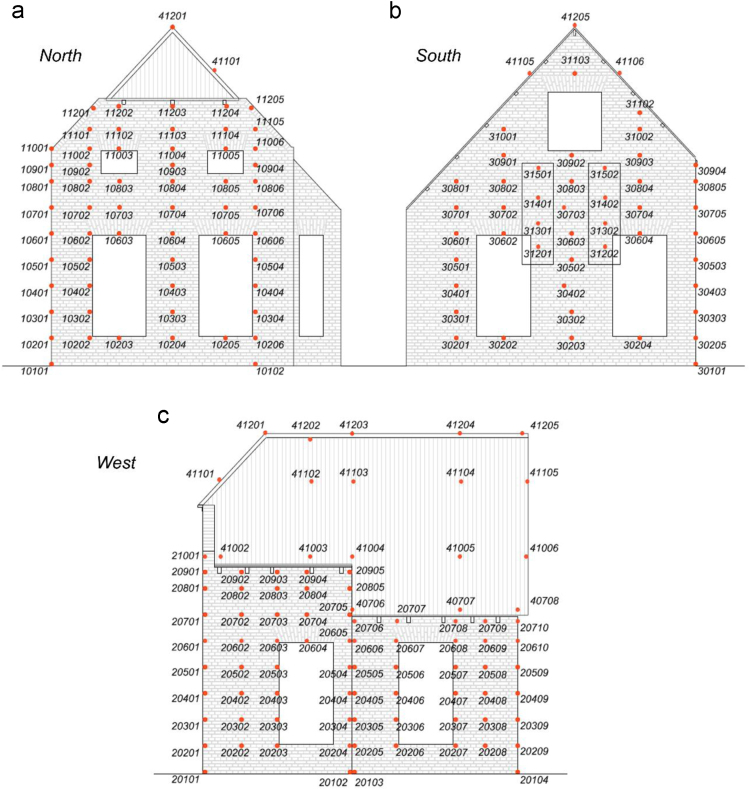
Location of the markers mounted on the specimen: (a) North view; (b) South view; (c) West view.

**Fig. 3:**
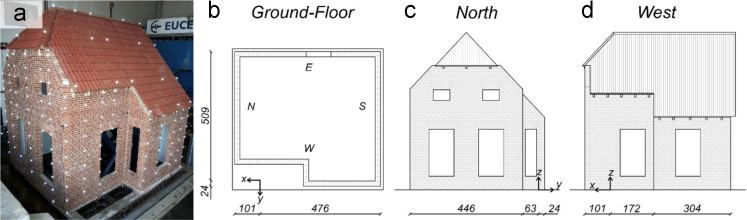
Reference coordinate system of the 3D optical motion-capture system: (a) location of targets on the specimen; (b) ground-floor plan; (c) North view; (d) West view. (units of cm).

A three-dimensional optical motion-capture system was also employed [3], [4]. Passive spherical markers coated with a retro-reflective material were placed on the West, North, and South façades. Fixed cameras recorded the *x*, *y* and *z* coordinates of the monitored points as they varied during the earthquake simulations, allowing to derive relative and total displacements of the building and local deformations of its components. For easier reference and data manipulation, each marker is identified by a code number, as shown on Fig. 2. The coordinate reference system is illustrated on Fig. 3.

**Fig. 4:**
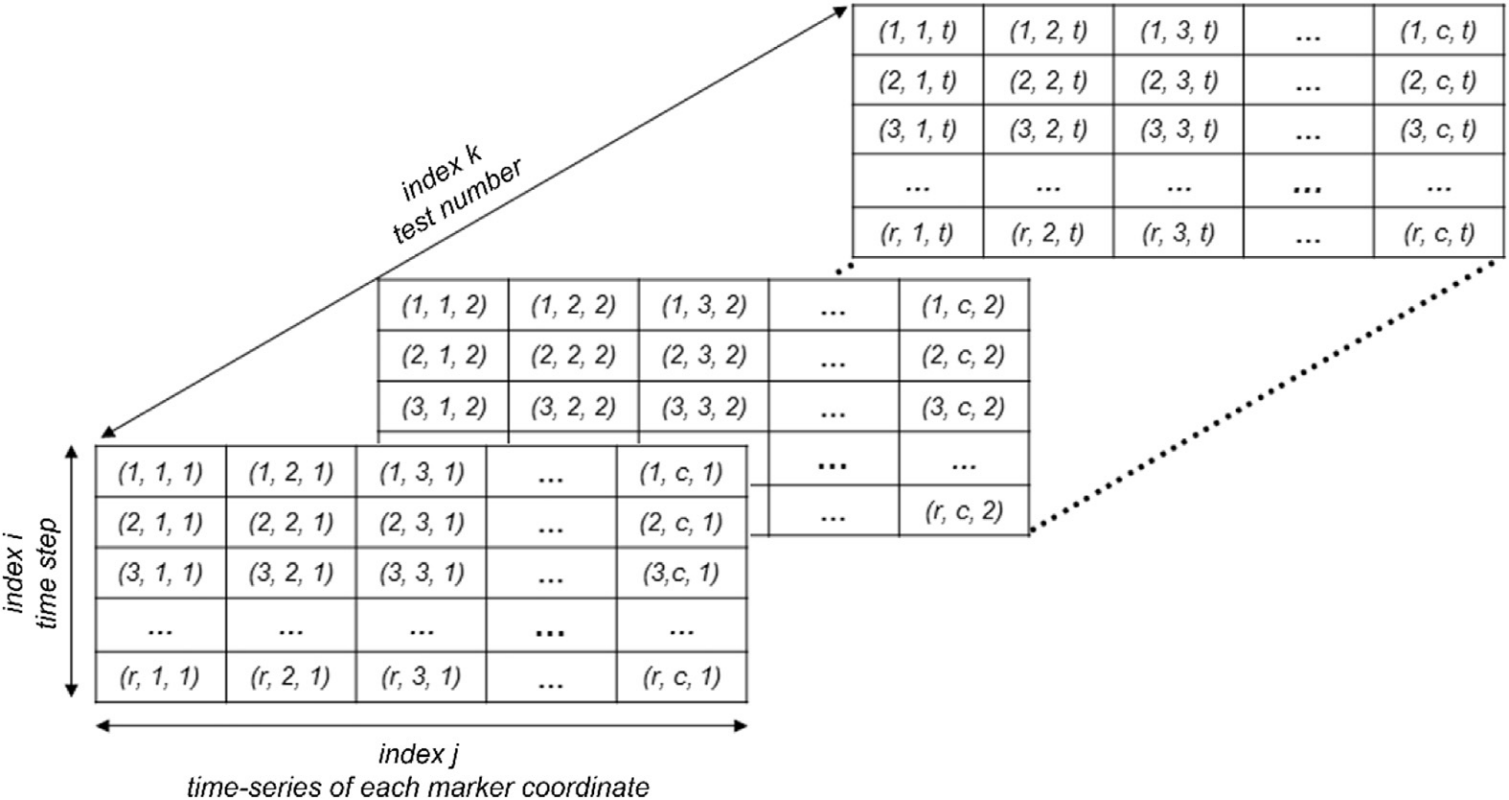
Structure of the data matrix “*EUC_BUILD_2_3DOpticalAcqData*”.

Recordings of the optical acquisition system are provided in a single *.mat* file, named “*EUC_BUILD_2_3DOpticalAcqData.mat*”, organized as a three-dimensional matrix with indices *i*, *j*, *k*. This matrix contains twelve two-dimensional matrices corresponding to the twelve tests. Each two-dimensional matrix has on its columns the time series of the three coordinates of the markers (in units of mm) based on the reference system shown on Fig. 3. The time series are synchronized with those obtained from potentiometers and accelerometers at sampling rate of 256 Hz. For reference to any element of the matrix, the indices follow these rules, as illustrated on Fig. 4:

i. The row index, *i*, varies from 1 to the length of the time series (5121 or 7681 components);
ii. The column index, *j*, varies from 1 to 583: column No. 1 contains the time at sampling rate of 256 Hz, while columns 2 to 583 contain the coordinate time history of each target along the *x*, *y* and *z* directions of the reference system;
iii. The third index, *k*, varies from 1 to 12 and indicates the number of test, according to Table 4.

**Table 4:** Optical acquisition data: content of 3D matrix “*EUC_BUILD_2_3DOpticalAcqData*”.

**Test No. - 3D matrix index*****k***	**Test name**	**Two-dimensional matrix sizes (rows No. × columns No.)**
1	SC1–25%	5121 × 583
2	SC1–50%	5121 × 583
3	SC1–100%	5121 × 583
4	SC1–150%	5121 × 583
5	SC2–50%	7681 × 583
6	SC2–100%	7681 × 583
7	SC2–150%	7681 × 583
8	SC2–200%	7681 × 583
9	SC2–250%	7681 × 583
10	SC2–300%	7681 × 583
11	SC2–400%	7681 × 583
12	SC2–200%-A^a^	7681 × 583

a A: test simulating an aftershock.

A complementary *.mat* file, called “*EUC_BUILD_2_3DOpticalAcqData_Labels.mat*”, provides a row vector for labelling the columns of each two-dimensional data matrix and relating them to the marker coordinates. Every marker ID number appears three times, each time followed by suffix “.1”, “.2” or “.3” to identify the *x*, *y* and *z* coordinates of the marker, respectively.

During the tests SC2 - 200%, 250% and 300%, the trajectories of some markers were not successfully tracked by the motion-detection system (“missing markers”): the corresponding time histories were substituted with “not-a-number” elements in the data matrix. Moreover, the recorded trajectories of a few markers (i.e., those at the N-E and S-E corners of the building) were interrupted when they displaced out of the viewing cone of the cameras (“ghost markers”): these gaps were filled with zeros in the post-processing of the data. Table 5 lists in black the ID numbers of the “missing markers” and the “ghost markers” for each dynamic test.

**Table 5:** Missing markers from the data matrix “*EUC_BUILD_2_3DOpticalAcqData*”.

Eight additional markers were mounted on the South façade only for the last two tests, SC2 - 400% and SC2–200%-A (aftershock): they are shown in boxes on Fig. 2 and listed in grey in Table 5.

The following MATLAB routine gives three examples of the use of the two provided*.mat* files:

**Table:** 

load EUC_BUILD_2_3DOpticalAcqData_Labels.mat;
load EUC_BUILD_2_3DOpticalAcqData.mat;
%% EXAMPLE 1: Give the time history of the x coordinate [in mm] of marker 20101 (test No. 11, SC2 - 400%)
coord_20101_x = EUC_BUILD_2_3DOpticalAcqData…
(:,find(EUC_BUILD_2_3DOpticalAcqData_Labels == 20101.1),11);
%% EXAMPLE 2: Give the time history of the y coordinate [in mm] of marker 20702 (test No. 5, SC2 - 50%)
coord_20702_y = EUC_BUILD_2_3DOpticalAcqData…
(:,find(EUC_BUILD_2_3DOpticalAcqData_Labels == 20702.2),5);
%% EXAMPLE 3: Give the time histories of the displacement of all markers [in mm]
Displ = EUC_BUILD_2_3DOpticalAcqData;
InitPos = EUC_BUILD_2_3DOpticalAcqData(1,:,:);
for k = 1:size(EUC_BUILD_2_3DOpticalAcqData,3)
for i = 1:size(EUC_BUILD_2_3DOpticalAcqData,1)
for j = 2:size(EUC_BUILD_2_3DOpticalAcqData,2)
Displ(i,j,k) = EUC_BUILD_2_3DOpticalAcqData(i,j,k) - InitPos (1,j,k);
end
end
end
